# Potential Chondroprotective Effect of *Artemisia annua* L. Water Extract on SW1353 Cell

**DOI:** 10.3390/ijms26051901

**Published:** 2025-02-22

**Authors:** Min Jung Kim, Ye Jin Yang, Ji Woong Heo, Jae-dong Son, Young Zoo You, Ju-Hye Yang, Kwang Il Park

**Affiliations:** 1 Department of Veterinary Physiology, College of Veterinary Medicine, Gyeongsang National University, Gazwa, Jinju 52828, Republic of Korea; minjung0102@gnu.ac.kr (M.J.K.); yang93810@gnu.ac.kr (Y.J.Y.); hujiw7806@gnu.ac.kr (J.W.H.); beeast0070@gmail.com (J.-d.S.); happyzoo0120@hanmail.net (Y.Z.Y.); 2 Korean Medicine (KM) Application Center, Korea Institute of Oriental Medicine, 70 Cheomdan-ro, Dong-gu, Daegu 41062, Republic of Korea; jjuhye@kiom.re.kr

**Keywords:** osteoarthritis, anti-inflammation, oxidative stress, *Artemisia annua* L., chondroprotection

## Abstract

Inflammation plays a critical role in the pathogenesis of osteoarthritis (OA). The objective of this study was to investigate the anti-inflammatory and chondroprotective properties of *Artemisia annua* L. water extract (AWE) following the induction of inflammation in cartilage cells (SW1353 cell) through the administration of interleukin-1 beta (IL-1β). We demonstrated significant antioxidant activity, as evidenced by elevated total phenolic and flavonoid content, in addition to robust free radical scavenging capacity, as assessed through DPPH (2,2-diphenyl-1-picrylhydrazyl) and ABTS (2,2′-azino-bis(3-ethylbenzothiazoline-6-sulfonic acid) assays. Its cytotoxic effects were assessed at a concentration of 200 μg/mL, where no cytotoxic signs were observed in SW1353 cells treated with IL-1β; the levels of reactive oxygen species (ROS) were notably reduced in a dose-dependent manner. The principal inflammatory markers, cyclooxygenase-2 (COX-2) and inducible nitric oxide synthase (iNOS), were significantly diminished by AWE treatment. AWE administration led to a dose-dependent reduction in the expression of key proteins involved in the mitogen-activated protein kinase (MAPK) and nuclear factor kappa-light-chain-enhancer of activated B cell (NF-κB) signaling pathways, ultimately resulting in a decrease in the release of matrix metalloproteinases (MMPs), specifically MMP-1 and MMP-13, which are known to contribute to cartilage degradation. Additionally, the levels of degraded collagen type II in the cartilage cells were restored. These findings suggest that reducing oxidative stress and inflammation, along with inhibiting activated MAPK and NF-κB signaling pathways, may ameliorate the progression of IL-1β-induced OA. Furthermore, a molecular docking analysis revealed a strong binding affinity of MMP-13, a critical mediator in the pathogenesis of OA. Six compounds were identified in AWE, corroborating its potential antioxidant and anti-inflammatory effects. Therefore, AWE may serve as a potentially useful therapeutic agent against OA by modulating inflammation-related mechanisms.

## 1. Introduction

Articular cartilage (AC) is a highly specialized tissue that covers the surfaces of joints [1], facilitating smooth movement with minimal friction and permitting pain-free joint motion. However, during the onset and progression of osteoarthritis (OA), AC is one of the tissues that can sustain significant damage [2]. OA is a common chronic inflammatory degenerative disorder that affects 10% of the global population [3]. In particular, AC has a very limited capacity for self-repair; therefore, once structural degeneration and damage begin, there is a high likelihood of progressive joint dysfunction and chronic pain [4]. OA is a complex condition that affects not only AC but also various tissues that constitute the joints including subchondral bone remodeling [5], meniscal degeneration [6], inflammation and fibrosis of the synovial membrane, and infrapatellar fat pad [7]. The prevalence of OA is influenced by a range of risk factors, such as occupational activities, sports participation, obesity, musculoskeletal injuries, and gender [8,9]. Even though OA significantly shortens the health span of many elderly individuals, a definitive method to completely halt its progression remains elusive. Current pharmacological management strategies primarily focus on symptom relief through analgesics, non-steroidal anti-inflammatory drugs (NSAIDs), and corticosteroid injections. However, these treatment methods provide only temporary alleviation of pain and are associated with potential side effects and toxicity when used long-term [10,11]. Consequently, there is an urgent need to develop compounds that can effectively manage OA while minimizing adverse effects.

A recent study has suggested that the usage of natural extracts is a potential candidate for the development of OA relief and treatment, without causing side effects or toxicity [12,13,14,15]. *Artemisia annua* L., an annual herb belonging to the Asteraceae family, has been noted in traditional medicine for its various effects [16]. The primary component, artemisinin, is known for its effectiveness in malaria treatment [17] and exhibits diverse biological activities, including anti-inflammatory [18], antioxidant [19], antimicrobial [20], and antitumor effects [21]. Furthermore, these effects have led to its use in the treatment of liver function improvement [22], immune enhancement, diabetes prevention, gastrointestinal disease amelioration, constipation alleviation [23], and atopic dermatitis [24] enhancement. However, the anti-inflammatory mechanisms and efficacy of artemisinin in treating OA, an inflammation-related disease, have not been reported. Comprehensive research is required to establish effectiveness and elucidate the mechanisms of these agents in the development of treatments for OA.

SW1353 cells are a cell line derived from human chondrosarcoma, exhibiting morphological and physiological characteristics similar to those of chondrocytes [25]. Notably, the expression of inflammatory cytokines and extracellular matrix-degrading enzymes, which play a crucial role in the pathological mechanisms of OA, has been reported to be induced in SW1353 cells [26]. This study has therefore selected the SW1353 cell line as an experimental model for investigating the mechanisms of chondrocyte inflammation in OA and demonstrates the potential therapeutic targeting of the water extract of *Artemisia annua* L. (AWE).

## 2. Results

### 2.1. Antioxidant Activity

**Figure 1:**
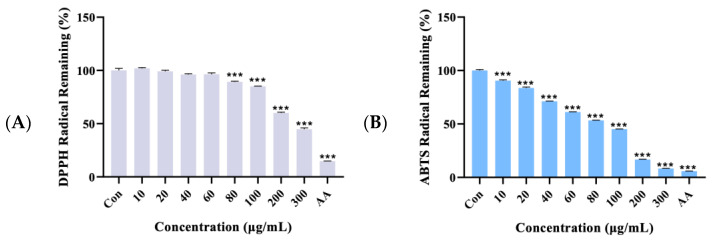
In vitro antioxidant activity of the AWE (**A**) DPPH (**B**) ABTS. Data represent the mean ± standard deviation (SD) of three independent experiments. *** *p* < 0.001 vs. the AWE-free control group. AA: 100 μg/mL ascorbic acid (positive control).

The total phenolic content of AWE was found to be 61.136 ± 0.74 mg gallic acid equivalents (GAE) per 100 g dry weight (d.w.) of plant material, while the total flavonoid content was 54.167 ± 1.705 mg quercetin equivalents (QE) per 100 g d.w. of plant material (Table 1). To evaluate the antioxidant capacity of AWE, DPPH (2,2-diphenyl-1-picrylhydrazyl) and ABTS (2,2′-azino-bis(3-ethylbenzothiazoline-6-sulfonic acid) assays were conducted to measure free radical remaining activities. Figure 1A,B illustrate the average residual percentages for DPPH and ABTS at different concentrations of AWE. The AWE demonstrated significant in vitro antioxidant activity. The DPPH radical remaining was observed at a concentration of 80 μg/mL, while the ABTS radical remaining was detected beginning at 10 μg/mL. At a concentration of 300 µg/mL (8.30925%) or higher, the ABTS antioxidant capacity at 100 μg/mL was approximately equal to that of the positive control ascorbic acid, which exhibited a value of 5.864%. These results confirm that AWE possesses a high antioxidant capacity.

### 2.2. Effect of AWE on the Activation of ROS in IL-1β Induced SW1353 Cell

**Figure 2:**
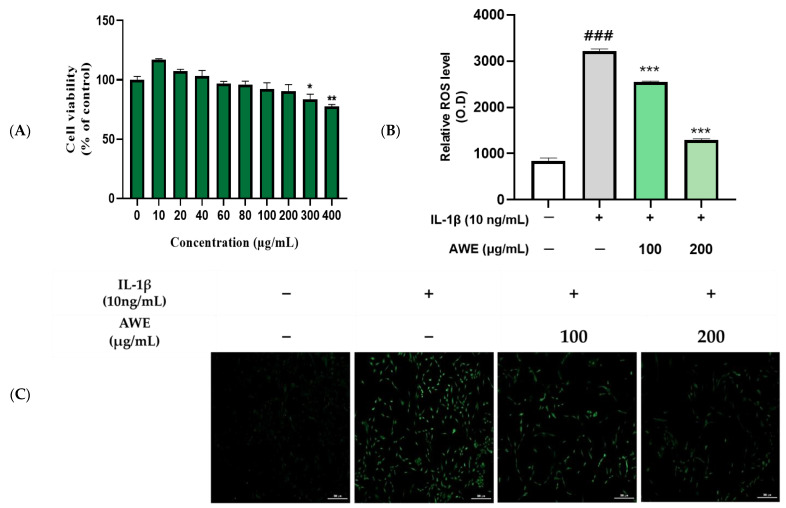
AWE treatment suppressed IL-Iβ-induced oxidative stress in SW1353 cell. (**A**) The cell viability assay following AWE treatment. Data represent the mean ± SD of three independent experiments. (**B**) Fluorescence micrograph and (**C**) fluorescence intensity of DCF-DA. Data represent the mean ± standard error of the mean (SEM). ^###^ *p* < 0.001 vs. untreated control. * *p* < 0.05; ** *p* < 0.01; *** *p* < 0.001 vs. IL-1β-treated group. Scale bar: 300 μm.

We treated the chondrocytes with AWE at different concentrations (0~400 μg/mL) for 24 h, respectively. The (3-(4,5-Dimethylthiazol-2-yl)-2,5-diphenyltetrazolium bromide) MTT assay demonstrated that the viability of chondrocytes was significantly diminished following treatment with concentrations of 300 and 400 μg/mL. AWE was administered for 24 h (Figure 1A), indicating that AWE at ≤200 μg/mL concentrations displayed no significant cytotoxic effects on chondrocytes. Hence, we selected AWE at 100 and 200 μg/mL to explore its role in OA progression. The oxidative stress levels in cells can be assessed using reactive oxygen species (ROS) measurement. Using DCF-DA, the ability of AWE to scavenge ROS was measured in IL-1β induced chondrocytic cells. The results showed a concentration-dependent decrease in fluorescence intensity when compared to the IL-1β induced group (Figure 2B). The IL-1β induced the brightest fluorescence signal, while the control group displayed the lowest fluorescence signal, indicating that IL-1β contributes to the accumulation of ROS, thereby inducing oxidative stress in chondrocytic cells (Figure 2C). This suggests that AWE can dose-dependently reduce ROS generation induced by IL-1β in chondrocyte cells, thereby exhibiting a protective effect against cellular oxidative stress damage.

### 2.3. Effect of AWE on the Activation of Inflammation in IL-1β-Induced SW1353 Cell

**Figure 3:**
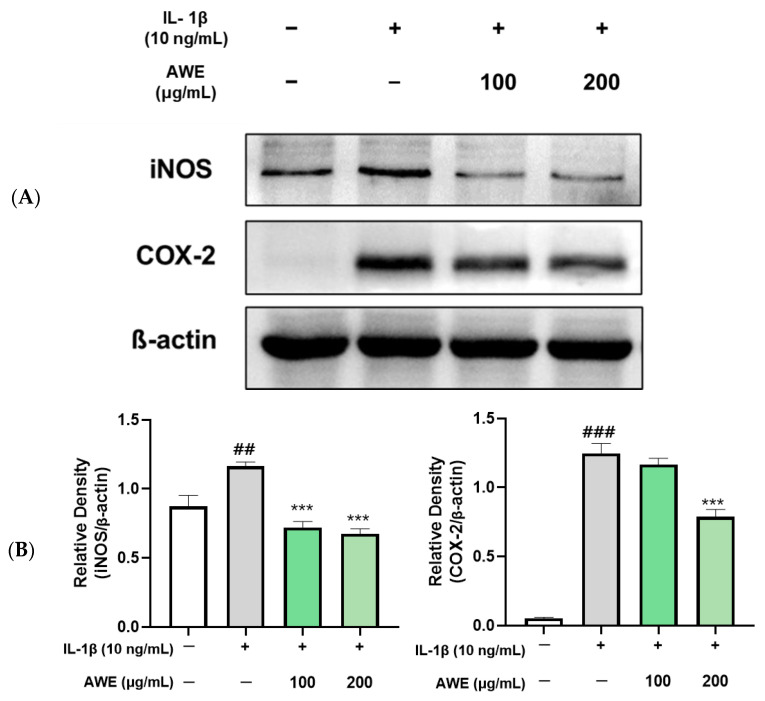
Effects of AWE on IL-1β- induced inflammation activation in the SW1353 cell. (**A**) The protein levels of iNOS and COX-2 were detected by Western blot with β-actin as internal reference. (**B**) quantification analysis. Data represent the mean ± SEM. ^##^ *p* < 0.01; ^###^ *p* < 0.001 vs. untreated control. *** *p* < 0.001 vs. IL-1β-treated group.

To investigate the effects of AWE on chondrocyte inflammation induced by IL-1β, chondrocytes were pretreated with AWE for 1 h at 100 and 200 μg/mL, respectively, and treated with 10 ng/mL IL-1β for 24 h. Western blot results confirmed the downregulatory effects of AWE on elevated inflammation-related protein levels caused by IL-1β, including inducible nitric oxide synthase (iNOS) and cyclooxygenase 2 (COX-2) (Figure 3). These results indicate that AWE suppresses inflammation in chondrocytes induced by IL-1β.

### 2.4. Effect of AWE on the Activation of MAPKs and NF-κB in IL-1β-Induced SW1353 Cell

**Figure 4:**
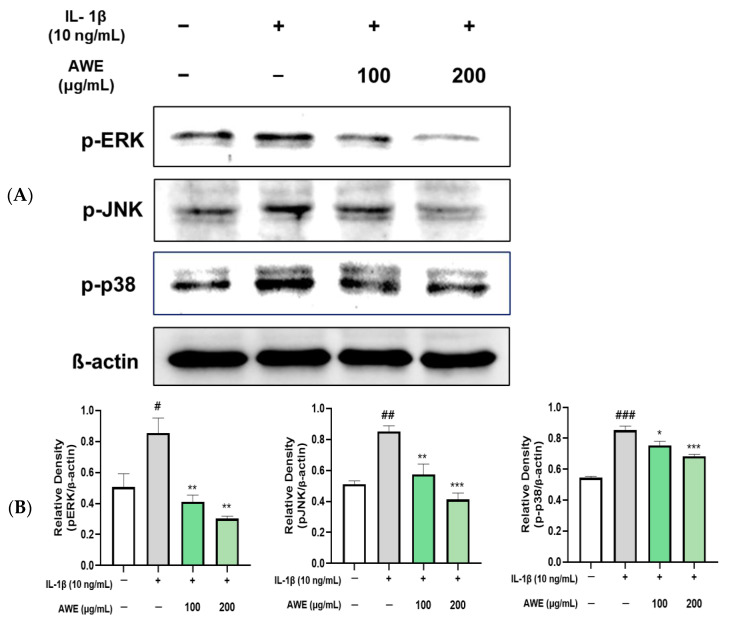
Effects of AWE on IL-1β-induced MAPKs activation in SW1353 cells. (**A**) The expression levels of the proteins p-ERK, p-P38, and p-JNK were assessed using Western blot analysis, with β-actin serving as the internal reference. (**B**) Quantification analysis. Data represent the mean ± SEM. ^#^ *p* < 0.05; ^##^ *p* < 0.01; ^###^ *p* < 0.001 vs. untreated control. * *p* < 0.05; ** *p* < 0.01; *** *p* < 0.001 vs. IL-1β-induced group.

The MAPK signaling pathway was significantly activated in chondrocytes following treatment with IL-1β. However, treatment with AWE significantly suppressed the phosphorylation of P38, ERK, and JNK in chondrocytic cells induced by IL-1β (Figure 4).

**Figure 5:**
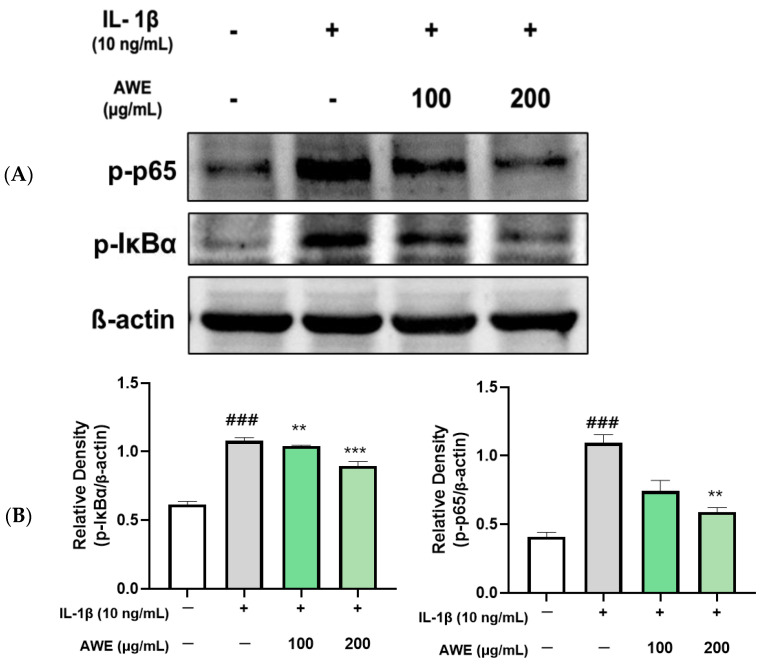
Effects of AWE on IL-1β- induced NF-κB activation in SW1353 cell. (**A**) The protein expression levels of p-NF-κB and p-IκBα were determined by Western blot with β-actin as the internal reference (**B**) quantification analysis. Data represents the mean ± SEM. ^###^ *p* < 0.001 vs. untreated control. ** *p* < 0.01; *** *p* < 0.001 vs. IL-1β-induced group.

We next evaluated the effects of AWE on the NF-κB pathway. The results indicated that IL-1β significantly increased phosphorylation of NF-κB and IκBα levels in chondrocytes. Meanwhile, AWE dramatically inhibited IL-1β-elevated phosphorylation of IκBα and NF-κB levels in a dose-dependent manner (Figure 5). Taken together, AWE inhibits the activation of MAPK and NF-κB signaling pathways in chondrocytes stimulated with IL-1β, an effect presumed to result from the suppression of oxidative stress and inflammation.

### 2.5. Effect of AWE on the Activation of Collagen II and MMPs in IL-1β-Induced SW1353 Cell

**Figure 6:**
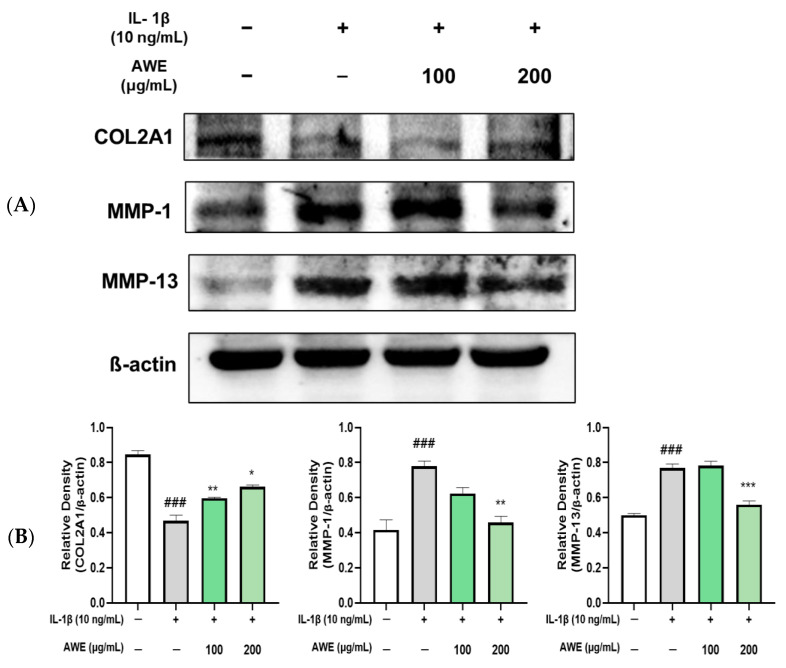
Effect of AWE on the expression of Type II collagen, MMP-1, and MMP-13 in the SW1353 cell. (**A**) The protein expression levels of COL2A1, MMP-1, and MMP-13 were analyzed using Western blot, with β-actin serving as internal reference. (**B**) Quantification analysis. Data represent the mean ± SEM. ^###^ *p* < 0.001 vs. untreated control. * *p* < 0.05; ** *p* < 0.01; *** *p* < 0.001 vs. IL-1β -induced group.

To investigate the effect of AWE pretreatment on the degradation of the extracellular matrix (ECM), we measured the protein levels of ECM-related genes in IL-1β-induced chondrocytes using Western blot analysis. Our results revealed that IL-1β treatment resulted in a reduction in collagen II expression while significantly enhancing the expression of MMP-1 and MMP-13. Notably, these alterations induced by IL-1β were substantially mitigated by AWE pretreatment, which correspondingly led to an increase in the expression of aggrecan and collagen II, alongside a decrease in MMP-1 and MMP-13 expression (Figure 6). These findings suggest that AWE effectively safeguards chondrocytes from ECM degradation induced by IL-1β.

### 2.6. Ultra Performance Liquid Chromatography–Quadrupole Time-of-Flight Mass Spectrometry (UPLC-TOF-MS/MS) Detection of AWE

**Figure 7:**
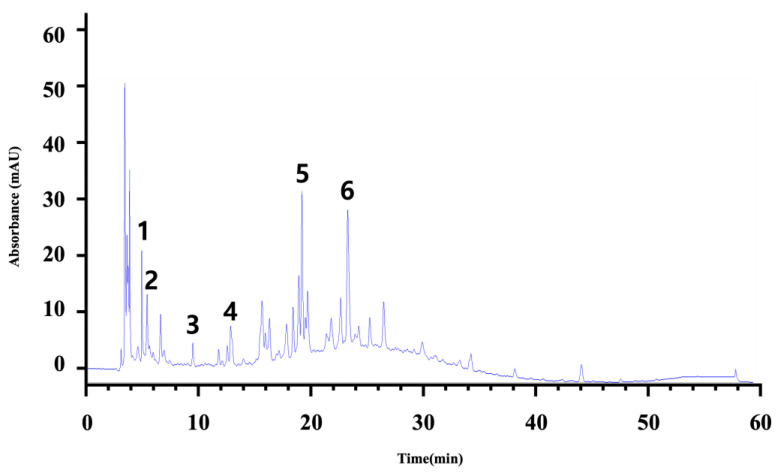
Tentative identification of the chemical components of the AWE obtained from the UPLC-TOF-MS/MS analysis. The six compounds nicotinic acid (Peak 1), uracil (Peak 2), phenylalanine (Peak 3), 3-indoleacrylic acid (Peak 4), coumarin (Peak 5), and isoscopoletin (Peak 6) are explained in terms of their chromatography characteristics.

The comprehensive Ultra Performance Liquid Chromatography (UPLC)-TOF-MS/MS analytical technique applied to the AWE yielded significant and noteworthy results, as illustrated in Figure 7 and Table 1. At a wavelength of 280 nm, the peaks of six compounds include nicotinic acid (Peak 1, C_6_H_5_NO_2_), uracil (Peak 2, C_4_H_4_N_2_O_2_), phenylalanine (Peak 3, C_9_H_11_NO_2_), 3-indoleacrylic acid (Peak 4, C_11_H_9_NO_2_), coumarin (Peak 5, C_11_H_8_O_2_), and isoscopoletin (Peak 6, C_10_H_8_O_4_), reflecting their diverse biological and chemical functions (Figure 7, Table 2).

**Figure 8:**
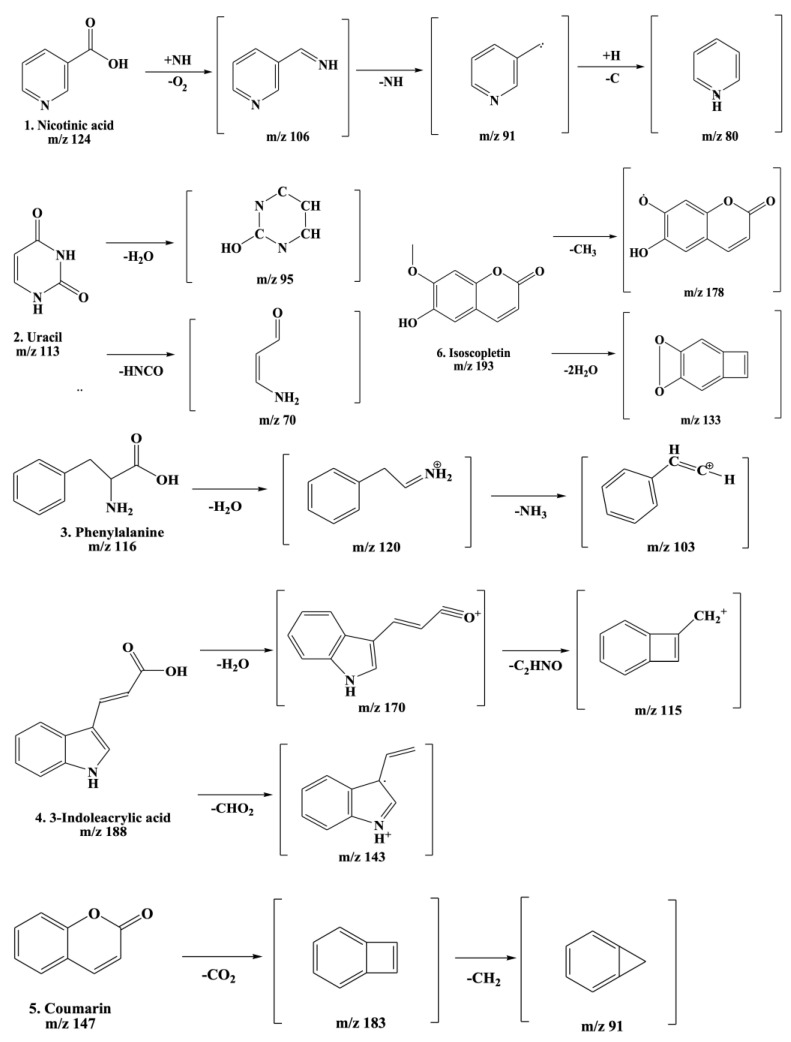
Fragmentation behaviors of nicotinic acid, uracil, phenylalanine, 3-indoleacrylic acid, coumarin, and isoscopoletin.

The fragmentation patterns displayed in Figure 8 are nicotinic acid (m/z 124,106,96,80) [27,28], uracil (m/z 113,96,70) [28], phenylalanine (m/z 116,120,103) [28], 3-indoleacrylic acid (m/z 188,170,143,155) [29], coumarin (m/z 147,103,91) [30], and isoscopoletin (m/z 193,178,133) [28]. Fragmentation patterns are analyzed to identify unknown compounds through structural breakdown and to determine molecular structures using mass spectrometry techniques like LC-MS and GC-MS.

### 2.7. Molecular Docking of AWE and MMP-13

**Figure 9:**
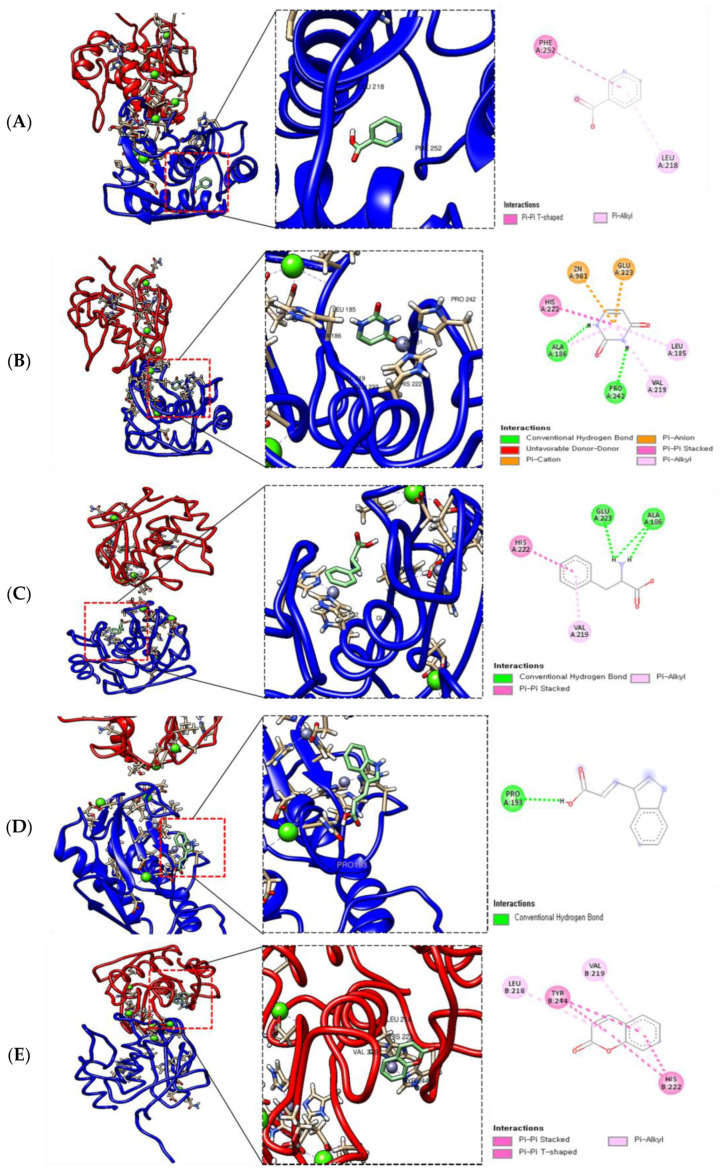

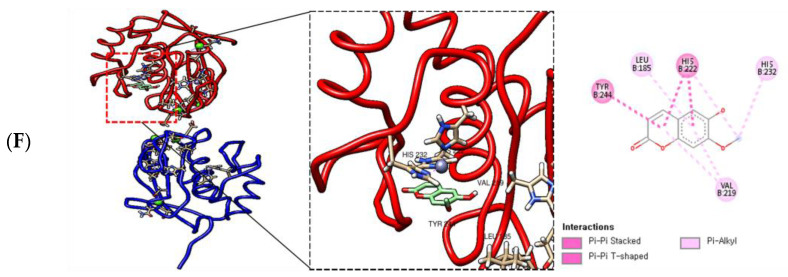
Molecular docking diagrams in two dimensions (right) and three dimensions (left) illustrating the interactions between nicotinic acid (**A**), uracil (**B**), phenylalanine (**C**), 3-Indoleacrylic acid (**D**), coumarine (**E**), Isoscopoletin (**F**), and the targeted protein MMP-13.

Binding energy refers to the energy released or absorbed when a drug molecule binds to its target. The stability of the drug–target interaction increases as the binding energy decreases. Therefore, a negative binding energy value usually indicates favorable binding. The results of molecular docking are presented in Figure 9. The different colors represent the chains in the 3D structure of MMP-13. Molecular docking analysis revealed compounds strong binding affinity. Molecular docking analysis revealed a strong binding affinity of MMP-13 with nicotinic acid (−5.0 kcal/mol), uracil (−4.9 kcal/mol), phenylalanine (−6.8 kcal/mol), and 3-Indoleacrylic acid (−5.4 kcal/mol), in addition to the strong binding affinity of MMP-13 with coumarine (−7.7 kcal/mol) and isoscopoletin (−7.7 kcal/mol) (Table 3).

## 3. Discussion

Oxidative stress plays an important role in the development and progression of OA. It occurs when the production of ROS exceeds the body’s antioxidant defenses. This imbalance disrupts intracellular signaling pathways associated with active inflammation [31], leads to cartilage cell death, inhibits cartilage matrix synthesis, and accelerates the progression of OA. Inflammation control in joint tissues and body fluids involve various factors, among which oxidative stress and inflammatory cytokines are closely related to the development and progression of OA [32]. The imbalance between oxidative stress and antioxidant defenses increases ROS production in cartilage and chondrocytes, accelerating cartilage degradation and exacerbating disease progression.

According to our study, AWE demonstrates excellent antioxidant properties, with high total flavonoid and total polyphenol content (Table 1 and Figure 1). Mitochondrial dysfunction and oxidative stress can lead to aging in bone and joint tissue cells, and the accumulation of chondrocyte senescence is strongly implicated in cartilage breakdown and OA progression [33]. Excessive production of free radicals in joint cartilage cells and a weakened antioxidant defense system are key factors in the development and progression of OA [24].

Therefore, regulating and reducing oxidative stress levels in joint cartilage can delay the progression of OA to some extent. In this study, ROS levels were significantly reduced by AWE, highlighting its antioxidant capacity in regulating oxidative stress (Figure 2). AWE functions as an antioxidant by minimizing oxidative stress, as evidenced in both the present and prior research [19], which may lead to reduced inflammation and potential relief of OA symptoms.

Continuous chronic inflammation in OA is driven by inflammatory mediators such as IL-1β, TNF-α, and IL-6. Patients with OA show significantly higher concentrations of IL-1β, IL-6, and TNF-α in their blood, synovial fluid, and cartilage tissue, as well as in other joint tissues such as the synovial membrane and infrapatellar fat pad compared to healthy individuals [34]. These elevated cytokines lead to the loss of proteoglycans and cartilage substrates, further degenerating joint cartilage. IL-1β plays a crucial role in the development of OA [35]. In this study, AWE significantly inhibited iNOS and COX-2 expressions, which were elevated by IL-1β (Figure 3). This indicates that AWE suppresses inflammatory reactions induced by IL-1β in cartilage cells and suggests that it may improve OA by reducing inflammation.

The ECM degradation process is regulated by several signaling pathways, among which MAPK and NF-κB are major contributors to cartilage degeneration and OA progression [36,37]. These pathways relay extracellular stimuli that activate the expression of genes involved in inflammation and ECM breakdown. The activation of the MAPK signaling pathway induces ECM degeneration, contributing to OA development. Similarly, NF-κB, which is normally sequestered in the cytoplasm by binding to Iκ-Bα, becomes activated when Iκ-Bα is phosphorylated. Once activated, the NF-κB subunit p65 translocates to the nucleus, regulating the expression of inflammatory mediators such as iNOS, COX-2, IL-1β, IL-6, and TNF-α, all of which drive OA progression [38,39]. Inhibiting NF-κB activity is crucial for reducing inflammation and preventing cartilage degeneration in OA [40]. Furthermore, since NF-κB signaling promotes ECM-degrading factors, targeting this pathway can prevent cartilage damage. In this study, AWE significantly reduced the phosphorylation of p65, Iκ-Bα, ERK, p38, and JNK, which were increased by IL-1β (Figure 4 and Figure 5). This demonstrates that AWE inhibits both MAPK and NF-κB signaling pathways in a cartilage cell model of OA, thereby exerting anti-inflammatory effects and regulating ECM degradation.

Prostaglandin E2, generated through nitric oxide and COX-2 induced by iNOS, has been reported to contribute to OA by promoting ECM degradation [41]. OA is closely linked to ECM degradation, which is controlled through complex pathways. MMPs play a key role in tissue remodeling, wound healing, vascular formation, and normal tissue turnover [42]. However, in OA, MMPs act as proteolytic enzymes that degrade essential components of bone and cartilage, contributing to joint and cartilage degeneration. Among MMPs, MMP-1 and MMP-13 are particularly implicated in OA progression, as their expression is significantly increased and is closely associated with cartilage degeneration [43,44]. The overproduction of MMPs induced by IL-1β inhibits the synthesis of type II collagen in cartilage cells. Type II collagen is critical for maintaining healthy cartilage structure, and its decreased synthesis and degradation lead to cartilage deterioration and progression of OA [45]. In this study, IL-1β increased MMP-1 and MMP-13 expression while reducing type II collagen expression (Figure 6). AWE effectively suppressed the expression of MMP-1 and MMP-13 and restored type II collagen levels, suggesting its potential to prevent cartilage degradation by inhibiting MMP activity induced by IL-1β.

The tentative identification of the AWE components was based on molecular weights, MS/MS fragmentation, as well as research data. Compounds were numbered according to their retention times (Figure 7 and Figure 8). MMP-13 is a target drug that plays a crucial role in the treatment of OA [46,47]. The energy interaction with AWE was analyzed. The molecular binding energy score revealed that the indicator substances of AWE, coumarine, and isoscopoletin exhibit the highest affinity at -7.7 kcal/mol (Figure 9 and Table 3). In particular, coumarin and isoscopoletin are well known for their anti-inflammatory effects, and recent studies have reported that coumarin positively influences the improvement of OA [48,49]. Based on these data, the chondroprotective effect of AWE is further supported by docking analyses and in vitro experimental results.

**Figure 10:**
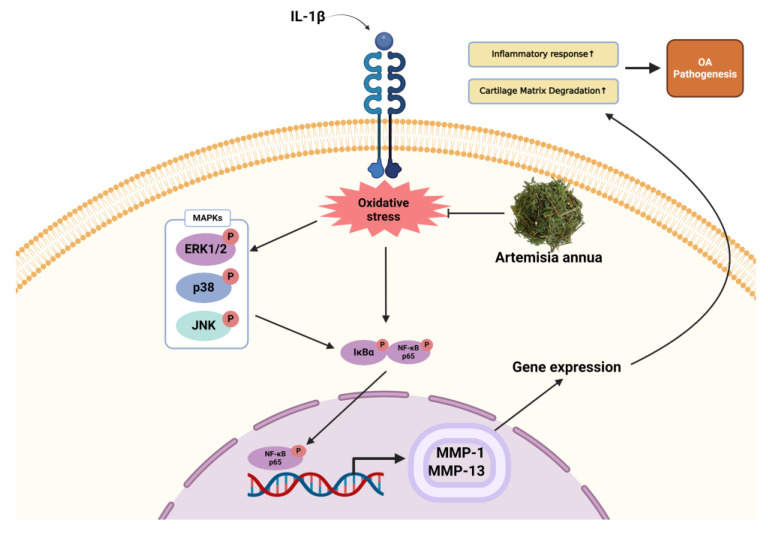
A schematic representation of possible mechanism by AWE anti-OA effect in SW1353 Human Chondrocytes. This image was created by BioRender (Kim, W. (2025) https://BioRender.com/w43c731) on 21 February 2025.

These results demonstrate the antioxidant and oxidative stress reduction effects of AWE in vitro, indicating its potential use as a therapeutic agent for relieving inflammation and providing chondroprotective effects. In this study, the anti-inflammatory effects of AWE were substantiated (Figure 10). However, a comparison with currently utilized anti-inflammatory medications for osteoarthritis (e.g., diclofenac, celecoxib) was not encompassed. Future research should integrate a comparative analysis with commonly employed anti-inflammatory medications to evaluate the relative therapeutic efficacy of AWE, while also expanding the investigation to animal models, such as the osteoarthritis mouse model, to validate its effects under more physiologically relevant conditions.

## 4. Materials and Methods

### 4.1. Plant Materials

*Artemisia annua* L. leaves were procured from the Yeong Cheon Oriental Herbal Market, located in Yeong Cheon, Korea. The *Artemisia annua* L. extracts (AWE) used in this study were prepared at 10 times the weight of warm water (60 °C) for 3 h. This process was repeated thrice, and the raw extract was then pooled, concentrated using a vacuum concentrator (EYELA, Tokyo, Japan, yield 8.34%), and stored at −20 °C. For individual experiments, 10 mg of water extracts from AWE was dissolved in 1 mL of distilled water and filtered through a 0.22-μm pore filter.

### 4.2. Total Phenolic and Total Flavonoid Content Determination

Total phenolic content was determined by a modification of the Folin–Ciocalteau method. Gallic acid (Sigma-Aldrich, St. Louis, MO, USA) solutions at a concentration of 1 mg/mL were prepared with distilled water and diluted by concentration for the preparation of calibration curves. A total of 500 μL of 2N Folin’s phenol reagent (Sigma-Aldrich, St. Louis, MO, USA) was added to 100 μL of gallic acid solutions of varying concentrations (6.25 μg/mL-300 μg/mL) and a 2 mg/mL sample. And then, 400 μL of 7.5% Na_2_CO_3_ was added and mixed. This was then distributed into 96-well plates and incubated for 1 h in the dark at room temperature. Absorbance was measured at 750 nm using a microplate reader (Agilent Technologies, Santa Clara, CA, USA). The total polyphenol content was quantified as mg GAE/g based on a calibration curve generated with gallic acid.

The determination of total flavonoid content is as follows: 1 mg/mL quercetin (Sigma-Aldrich, St. Louis, MO, USA) solution was prepared with 100% methanol (Daejung Chemicals, Siheung, Republic of Korea) and diluted to various concentrations to generate a calibration curve. To 100 μL of quercetin solutions of various concentrations (6.25 μg/mL-200 μg/mL) and a 2 mg/mL sample, 860 μL of 80% ethanol, 20 μL of 10% AlCl_3_, and 20 μL of 1M potassium acetate (BIOSTEM, Seoul, Republic of Korea) were added and mixed, and then distributed into a 96-well plate. The reaction was then incubated for 40 min at room temperature, and the absorbance was measured at 415 nm using a microplate reader. The total flavonoid content was determined using a calibration curve with quercetin as standard and expressed as mg QE/g.

### 4.3. Antioxidant Capacity Analysis

In vitro antioxidant capacities were assessed using two different methods: the DPPH remaining activity assay and ABTS^•+^ (Thermo Fisher Scientific, Waltham, MA, USA) remaining activity assay. The DPPH assay was performed using a solution that was diluted with pure methanol (Daejung Chemicals, Siheung, Republic of Korea) to adjust the absorbance to 1.00 ± 0.10 at 517 nm before use. Samples were prepared by diluting the stock solution to various concentrations using pure methanol. A mixture of 100 μL of each sample at varying concentrations and 100 μL of the diluted DPPH solution was incubated in the dark at 37 °C for 30 min. Post-incubation, the absorbance was measured at 517 nm using a microplate spectrophotometer (Agilent Technologies, CA, USA) to assess the remaining DPPH radical levels. In the case of the control group, 100 μL of pure methanol replaced the sample.

The ABTS assay was performed as follows: 7.4 mM ABTS (Sigma-Aldrich, St. Louis, MO, USA) and 2.6 mM potassium persulfate (Sigma-Aldrich, St. Louis, MO, USA) were dissolved in distilled water and combined in a 1:1 ratio. This mixture was subsequently incubated in the dark at room temperature overnight for the generation of ABTS^+^ radicals. The resultant ABTS^+^ solution was diluted with distilled water to adjust its absorbance to 0.7 ± 0.02 at 734 nm. In a 96-well plate, 100 μL of each diluted sample was combined with 100 μL of the diluted ABTS^+^ solution, and the absorbance at 734 nm was measured immediately using a microplate spectrophotometer. Distilled water served as the control group instead of the sample. Ascorbic acid (Sigma-Aldrich, St. Louis, MO, USA) served as the positive control in both the DPPH and ABTS assays.

### 4.4. Cell Culture and Viability Assay

Human-derived chondrocyte SW1353 cells were obtained from ATTC and cultured in a medium consisting of DMEM (Gibco, Carlsbad, CA, USA) supplemented with 10% (v/v) Fetal Bovine Serum (Gibco, Carlsbad, CA, USA), 100,000 units/L of penicillin (Gibco, Carlsbad, CA, USA), and 100 mg/L of streptomycin (Gibco, Carlsbad, CA, USA). The cells were maintained in a humidified CO_2_ incubator at 37 °C. Once the cells reached approximately 80% confluence in the culture plates, they were washed with phosphate-buffered saline (BioSeSang, Seoul, Korea) (pH 7.4) and subsequently treated with 0.25% trypsin-2.65 mM EDTA (Gibco, Carlsbad, CA, USA) for subculturing. The culture medium was replaced every two days.

The cells were cultured in a (1 × 10^4^) well, plated on 96 well plates, and incubated for 24 h. After incubation with AWE at various concentrations for 1 h, the cells were stimulated with IL-1β (NKMAX, Seongnam, Republic of Korea) (10 ng/mL) for 24 h. For determining cell viability, after all treatments, the medium was removed, and MTT (Duchefa Biochemie, Haarlem, The Netherlands) solution was added to each well. Following incubation in the dark for 2 h, the supernatant was replaced with an equal volume of dimethyl sulfoxide to dissolve blue formazan crystals. The absorbance of the samples was determined at 570 nm using a BioTek H1 microplate reader (Agilent Technologies, CA, USA).

### 4.5. ROS Assay

SW1353 cells were cultured a 6-well plate 5 × 10^5^ cells/well and allowed to stabilize for 24 h 37 °C, in 5% CO_2_ conditions. Afterward, ROS stained using ROS-ID total ROS detection kit according to the manufacturer’s protocol (ENZ-51011, Enzo Life Sciences, Plym, MA). DCF-DA (Thermo Fisher Scientific, Waltham, MA, USA) was introduced and left to incubate for 1 h before being observed under a BioTex Fluorescence/multimode microplate reader (Agilent Technologies, Santa Clara, CA, USA) and BioTex Cytation 7 imaging multimode reader (Agilent Technologies, Santa Clara, CA, USA).

### 4.6. Western Blotting

SW1353 cells (1 × 10^6^) per well were plated on 60mm Petri dishes. Cells were washed with PBS (BioSeSang, Seoul, Republic of Korea) and lysed with radioimmunoprecipitation assay (RIPA) lysis buffer (Thermo Fisher Scientific, Waltham, MA, USA) containing protease and phosphatase inhibitors (GENDEPOT, Barker, TX, USA) 24 h after treatment. The supernatant was extracted, and protein concentrations were quantified using the BCA Protein Assay Kit (BioMax, Seoul, Republic of Korea). A total of 20 μg of protein was equally separated on a 10% SDS-PAGE gel. Proteins were transferred to polyvinylidene fluoride membrane. The membranes were blocked for 2 h with 5% skimmed milk (MBCell, Seoul, Republic of Korea) in TBS-T buffer and then incubated with primary antibodies diluted in TBS-T, including COX-2 (#12282, 1:1000), iNOS (#13120, 1:1000), p-ERK (#9101, 1:1000), p-p38 (#8690, 1:1000), p-JNK (#4668, 1:1000), p-NF-κB (#3031, 1:1000), p IκBα (#2859, 1:1000), MMP-1 (#54376, 1:1000), MMP-13 (#69926, 1:1000), COL2A1 (#43306, 1:500) (Cell Signaling Technology, Danvers, MA, USA), and β-actin (#ATGA0570, 1:1000, NKMAX, Korea) overnight at 4 °C. After being washed in TBS-T buffer, the membranes were incubated with secondary antibodies diluted in TBS-T for 2 h at 4 °C. Thermo Scientific SuperSignal West Pico PLUS Chemiluminescent Substrate was used for protein detection (Thermo Fisher Scientific, Waltham, MA, USA). ChemiDoc imaging equipment (Shenhua Science Technology, Hangzhou, China). The loading control utilized was the β-actin protein, and quantification of the Western blot images was conducted using the Image J software 1.54 K (U.S. National Institutes of Health, Bethesda, MD, USA).

### 4.7. UPLC-QTOF-MS Conditions

AWE was analyzed utilizing a Nexera XS UPLC system (Shimadzu; Kyoto, Japan) coupled with an X500R QTOF-MS (SCIEX ExionLC AD system; Framingham, MA, USA) equipped with an electrospray ionization source (ESI). Ion source gases 1 and 2 were each set at 50 psi, while the ion source temperature was sustained at 550 °C. A Pronto SIL 120-5-C18 SH column (150 × 4.6 mm, 5 μm) (Bischoff chromatography, Leonberg, Germany) was employed for UPLC, with the column oven maintained at a temperature of 35 °C. The flow rate was established at 0.5 mL/min, and the injection volume was 3 μL. The mobile phase consisted of 0.1% formic acid (Supelco, Bellefonte, PA, USA) in H_2_O (solution A) and 0.1% formic acid in acetonitrile (Sigma-Aldrich, St. Louis, MO, USA) (solution B). The gradient elution was configured as follows: from 0 to 5% solution B for 0–2 min; from 5 to 50% solution B for 2–40 min; from 50 to 90% solution B for 40–55 min; and from 90 to 5% solution B for 55–54 min. The identification of compounds via QTOF/MS was conducted in both positive (ESI at 5500 V) ion modes. The mass range for TOF MS and MS/MS was designated from 100 to 1500 Da and from 50 to 1500 Da, respectively. The declustering potential (DP) and collision energy (CE) for TOF MS/MS were set to 80 V and 35 ± 15 V in positive ion mode. Data processing was performed using a non-targeted screening methodology combined with a Purity Library Search algorithm for the identification of compounds. The parameters of the algorithm included a precursor mass tolerance of ±0.02 Da. Formula Finder was utilized to identify naturally occurring compounds within the following elemental composition limits: C (up to 100), H (up to 200), N (up to 20), O (up to 50), and S (up to 5). Data acquisition and compound identification were conducted using SCIEX OS software version 3.0.0.3339.

### 4.8. Molecular Docking Analysis

The protein structure from the Protein Data Bank (PDB) was accessed in order to conduct a molecular docking analysis via the search function on the website (https://www.rcsb.org/, accessed on 20 August 2023) (PDB code; 3KEJ, MMP-13). From PubChem (https://pubchem.ncbi.nlm.nih.gov, accessed on 10 May 2023), the 3D compound structures of nicotinic acid (Compound CID: 938), uracil (Compound CID: 1174), phenylalanine (Compound CID: 6140), 3-Indoleacrylic acid (Compound CID: 5375048), coumarine (Compound CID: 323), and Isoscopoletin (Compound CID: 69894) were downloaded. Docking analysis was conducted using AutoDock Vina with its default parameters. The exhaustiveness was adjusted to 8, the number of binding modes was set to 9, and the maximum allowable energy difference was defined as 3 kcal/mol. The docking results were visualized using PyMOL and Discovery Studio. Binding affinity was evaluated based on calculations of total intermolecular energy and the predicted binding free energy [50]. All experiments were conducted in triplicate, with a root mean square deviation (RMSD) of ≤2 Å.

### 4.9. Statistical Analysis

The data were expressed using the mean, standard deviation, and standard error of the mean. The data were analyzed using GraphPad Prism software (version 9.3.1; GraphPad Software, Inc., Boston, MA, USA). They were analyzed with a one-way factorial analysis of variance (ANOVA) to ascertain significant differences among them. After that, Dunnett’s multiple comparison tests were performed. A *p*-value of <0.05 was considered statistically significant (denoted as ^#^ *p* < 0.05, ^##^ *p* < 0.01, ^###^ *p* < 0.001 compared to the untreated control group; and * *p* < 0.05, ** *p* < 0.01, *** *p* < 0.001 compared to the IL-1β- treated group).

## 5. Conclusions

AWE exhibits chondroprotective properties by inhibiting the MAPK and nuclear factor kappa-light-chain-enhancer of NF-κB signaling pathways, resulting in the downregulation of inflammatory mediators such as inducible iNOS and COX-2, as well as MMP-1 and MMP-13, alongside the preservation of cartilage matrix components. These findings indicate that AWE may have therapeutic potential for the enhancement of joint and cartilage health.

## Figures and Tables

**Table 1:** Total phenolic and flavonoid contents of AWE.

Items		Concentration
AWE	Total polyphenol (GAE mg/g)	61.136 ± 0.74
	Total flavonoid (QE mg/g)	54.167 ± 1.705

**Table 2:** Metabolite profiling of *AWE* via UPLC-TOF-MS/MS in the positive ion mode.

Peak No.	Retention Time (min)	Compound (Molecular Formula)	MS/MS Fragment (m/z)	Chemical Structures (PubChem CID)
1	4.87	Nicotinic acid (C_6_H_5_NO_2_)	124,106,96,80	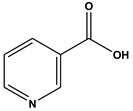 (938)
2	5.34	Uracil (C_4_H_4_N_2_O_2_)	113,96,70	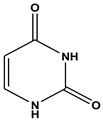
3	9.46	Phenylalanine (C_9_H_11_NO_2_)	166,120,103	(1,174) 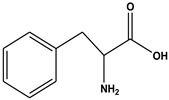 (6,140)
4	12.92	3-Indoleacrylic acid (C_11_H_9_NO_2_)	188,170,143,155	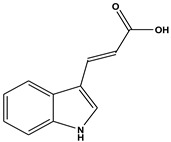 (5,375,048)
5	19.32	Coumarin (C_11_H_9_NO_2_)	147,103,91	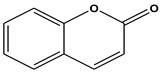 (323)
6	23.46	Isoscopoletin (C_10_H_8_O_4_)	193,178,133	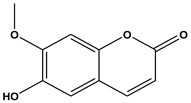
				(69,894)

**Table 3:** Molecular docking studies of compounds *AWE* with MMP-13 and their binding energy.

Binding Ligand	Amino Acid Residue That Interacts	Docking Score
Nicotinic acid	MET 252A, LEU 218A	−5.0 kcal/mol
Uracil	ALA 186A, HIS 222A, ZN 901A, GLU 223A, LEU 185A, VAL 219A, PRO 242A, ALA 186A	−4.9 kcal/mol
Phenylalanine	HIS 222A, GLA 223A, ALA 186A, VAL 219A	−6.8 kcal/mol
3-Indoleacrylic acid	PRO 193A	−5.4 kcal/mol
Coumarine	LEU 218B, TYR 244B, VAL 219B, HIS 222B	−7.7 kcal/mol
Isoscopoletin	TYP 244B, LEU 185B, HIS 222B, HIS 232B, VAL 219B	−7.7 kcal/mol

## Data Availability

The original contributions presented in this study are included in the article; further inquiries can be directed to the corresponding author(s).
